# Introduction of respiratory virus point-of-care testing at multi-site front-door admission wards

**DOI:** 10.1016/j.infpip.2026.100587

**Published:** 2026-08-28

**Authors:** Alzahraa Abbas, Ijeoma N. Okoliegbe, Noha El Sakka

**Affiliations:** a Department of Medical Microbiology and Virology, NHS Grampian, Aberdeen, UK; b School of Medicine, Medical Sciences and Nutrition, University of Aberdeen, Aberdeen, UK

**Keywords:** POCT, Respiratory virus, Front-door admission, Rapid PCR, Acute wards

## Abstract

**Background:**

During the coronavirus disease 2019 pandemic, rapid point-of-care testing (POCT) multiplex polymerase chain reaction assays for severe acute respiratory syndrome coronavirus 2 (SARS-CoV-2) and influenza A/B viruses were introduced at four front-door admission sites.

**Aim:**

To provide timely diagnosis of respiratory infections, help inform clinical decision-making, and potentially support patient flow and reduce the risk of hospital-acquired infections. This study also investigated the impact on laboratory testing demand as a proxy for workload, and the propriety of POCT use by ward staff.

**Methods:**

Respiratory samples tested by the laboratory or by POCT between January 2022 and June 2024 were analysed retrospectively to assess testing demand, rate of positivity, turnaround time (TAT), and staff competency in the use of POCT.

**Results:**

Of the 42,073 respiratory samples received, 54% (*N*=22,664) were tested by the virology laboratory and 46% (*N*=19,409) by POCT. Among adults, 44% of influenza-A-positive samples and 63% of SARS-CoV-2-positive samples were from patients aged ≥65 years. Among children, 69% of influenza-A-positive samples and 72% of SARS-CoV-2-positive samples were from patients aged 0–4 years. Only 4.3% of POCT results were invalid, suggesting acceptable operational performance in routine use. The time to result was significantly shorter for POCT compared with laboratory testing for influenza A/B (approximately 20 min vs 1319 min; *P*<0.05) and SARS-CoV-2 (approximately 20 min vs 418 min; *P*<0.05), reflecting more rapid availability of results for clinical decision-making.

**Conclusion:**

Introduction of rapid POCT at front-door admission was associated with a reduction in laboratory testing volume and shorter analytical TAT, which may support timely clinical management.

## Introduction

Acute respiratory tract infections (ARTIs) represent the most prevalent infections across all age groups, and pose a significant public health challenge due to their high transmissibility and associated morbidity and mortality rates [1,2]. In March 2025, World Health Organization (WHO) data showed 778 million cases of coronavirus disease 2019 (COVID-19), with 7.1 million deaths globally [3]. Additionally, WHO has reported one billion cases of seasonal influenza annually, with 3–5 million severe cases leading to between 290,000 and 650,000 respiratory-related deaths each year [4]. The incidence of morbidity and mortality due to ARTIs is highest among paediatric patients (age 0–5 years) and elderly patients (age ≥65 years) [[4], [5], [6], [7]], indicating their higher susceptibility to hospital admission and mortality [8,9]. The majority of deaths in children aged <5 years with influenza-related lower respiratory tract infections are in developing countries [4].

ARTIs are caused by both bacteria and viruses. They present with similar clinical symptoms such as cough, fever, nasal congestion, and respiratory failure in severe cases. Differentiating between bacterial and viral infections based on clinical features alone is challenging [10,11], and this may impact therapeutic decision-making, including use of antibiotics [12]. There is also a need for a fast turnaround time (TAT) for test results to allow timely diagnosis and management [13]. Laboratory testing includes several time-demanding steps, including sample transport, sample preparation, analysis, verification, and authorization of results [13]. This could lead to a long TAT that results in delayed diagnosis and clinical management, unnecessary prescription of antibiotics, and increased risk of antimicrobial resistance [[12], [13], [14]]. The use of point-of-care testing (POCT) is a way to overcome these challenges. POCT provides a rapid and sensitive diagnostic pathway that may support improved patient care and more timely clinical decision-making. It may also support antimicrobial stewardship and infection control practices where timely results are required [15,16].

POCT involves the use of diagnostic tests near the site of patient care, performed by non-laboratory healthcare workers. One of the principal advantages of POCT is its rapid TAT, compared with conventional laboratory-based polymerase chain reaction (PCR) methods which involve additional pre-analytical steps such as transportation and batch processing. Also, POCT for respiratory viruses has demonstrated accurate diagnostic performance, comparable with laboratory PCR assays [17,18]. In 2006, WHO established the ASSURED criteria for POCT, emphasizing the need for diagnostics that are affordable, sensitive, specific, user-friendly, rapid and robust, equipment-free, and deliverable to end-users [19]. The expansion of POCT has accelerated in recent years, particularly in response to the COVID-19 pandemic, leading to significant advancements in diagnostic technology and broader clinical uptake. This evolution has created new opportunities for research into the impact of POCT implementation across various hospital settings, particularly in high-throughput, front-door admission wards where rapid diagnostic support is critical.

Implementation of POCT in front-door admission wards, particularly in emergency departments (EDs) and acute medical assessment units (AMAUs) and paediatric assessment units, has gained increasing attention due to the high patient throughput in these settings. These environments require timely diagnostic support to inform clinical decisions [20]. However, most studies have been conducted in single-centre settings, and may not reflect implementation across multiple clinical environments [11,12,[21], [22], [23], [24], [25], [26], [27], [28], [29], [30], [31], [32]]. As such, this multi-site study was conducted to evaluate the implementation of respiratory POCT across four front-door admission wards. The utilization of POCT, impact of laboratory testing volume, and staff training and competency in POCT use were assessed, and TAT was compared between POCT and laboratory-based PCR testing.

## Methods

### Study design and setting

This study was a multi-site evaluation of the introduction of respiratory POCT at NHS Grampian's Aberdeen Royal Infirmary (ARI), Royal Aberdeen Children Hospital (RACH), and Doctor Gray's Hospital (DGH), covering different geographical areas and population types. ARI is a tertiary referral centre serving Northeast Scotland, and includes both an ED and an acute medical initial assessment unit (AMIA). RACH is co-located with ARI. DGH is a district general hospital in Moray, Elgin, located approximately 3 h from ARI, with routine thrice-daily transport of samples to the central virology laboratory at ARI. A retrospective analysis was conducted between January 2022 and June 2024 across four front-door admission areas: the ED and AMIA at ARI, the paediatrics assessment unit (PAU) at RACH, and the AMAU at DGH. As this work constituted a service evaluation, ethical approval was not required.

During the study period, respiratory samples were tested for SARS-CoV-2 and influenza A/B using either POCT or laboratory-based PCR testing. Testing was performed according to an agreed diagnostic pathway for respiratory virus testing. POCT was used where rapid results were required in line with this pathway, while other samples were processed through the laboratory pathway. Although testing followed an agreed pathway, differences between patient groups undergoing POCT and laboratory testing could not be fully excluded, consistent with the use of POCT based on clinical need. Patients with negative POCT results but ongoing clinical suspicion were managed according to standard clinical pathways, including follow-up laboratory testing where appropriate.

For the laboratory pathway, respiratory samples were processed daily using batch multiplex PCR assays on commercial diagnostic platforms. POCT was performed using the Cobas Liat system (Roche, Basel, Switzerland), which provided SARS-CoV-2 and influenza A/B results in approximately 20 min. Instruments were located within clinical areas with appropriate infrastructure for secure access, temperature-controlled storage, and electronic connectivity.

The wards were responsible for stock control, and kits were supplied by the laboratory on request. The set-up included securing information technology (IT) connectivity for the POCT instruments, so that results were transferred automatically to the electronic patient record, laboratory information management system (LIMS), infection control IT network, and public health notification system. The interface with LIMS allowed laboratory staff to pick up POCT results and avoid duplication of testing.

Ward staff were trained in the use of POCT through a structured programme that included supervised practice and formal competency assessment prior to independent use. Only staff who demonstrated competency were authorized to perform testing using individual log-in credentials. Training covered sample handling, test operation, and waste disposal in accordance with local policies. Ongoing competency was supported through quality control procedures, routine monitoring, and designated ward-based POCT champions responsible for maintaining training records and liaising with the laboratory. This structured training and governance framework was implemented to ensure and maintain operator competency in routine clinical use.

### Data collection and statistical analyses

Retrospective data were collected, including patient demographics (age and sex), location, specimen type, test type, results, and timestamps for sample receipt and result reporting.

Laboratory TAT was defined as the time from sample receipt (excluding pre-laboratory transport time) at the laboratory to result reporting. POCT turnaround time was defined as the time to result availability to the treating clinician following processing on the POCT device (approximately 20 min). While both pathways involve pre-analytical processes, these are considerably shorter for POCT due to near-patient testing, on-demand operation without batch processing, and delivery by a single operator during the clinical assessment. Consequently, laboratory TAT reflects a broader sequence of pre- and post-analytical steps, whereas POCT TAT predominantly captures the near-patient testing process within the clinical workflow.

Age subgroup analysis was performed using predefined categories: 0–4, 5–18, 19–44, 45–64, and ≥65 years. POCT was applied based on clinical need in accordance with an agreed diagnostic pathway, rather than targeting specific age groups. As such, age was not used as a predefined selection criterion for POCT allocation, although differences in case-mix between POCT and laboratory testing pathways cannot be fully excluded.

The proportion of valid and invalid POCT results was assessed as a pragmatic proxy indicator of operational performance and user competency in routine clinical practice; however, it is recognized that invalid results may also arise from device-, sample- or process-related factors, and temporal trends in invalid POCT rates were not formally analysed due to the relatively small number of invalid results.

Percentages for subgroup analyses were calculated using the relevant total number of samples within each group as the denominator. In addition, a comparative evaluation of test TAT was undertaken between POCT and central laboratory testing pathways to assess differences in diagnostic efficiency.

Statistical analysis was conducted using non-parametric comparative methods. As TAT data were not normally distributed, results have been presented as median and interquartile range, and comparisons were performed using the Mann–Whitney *U*-test. A *P-*value <0.05 was considered to indicate significance.

## Results

During the study period (January 2022–June 2024), a total of 42,073 respiratory samples were analysed using both POCT and virology laboratory pathways. Of these, 46% (*N*=19,409) were tested by POCT and 54% (*N*=22,664) were tested by the laboratory.

Among POCT samples, six were excluded from age-based analyses due to missing age data, while in the laboratory pathway, six samples were excluded from sex-based analyses due to unidentified sex. Laboratory samples included 54.68% (*N*=11510) males and 53.05% (*N*=11148) females, while POCT included 45.32% (*N*=9541) males and 46.95% (*N*=9868) females (Table I).

**Table I:** Demographics of samples tested using point-of-care testing (POCT) in front-door settings compared with laboratory testing during the study period (January 2022–June 2024)

Criteria	Testing	Paediatrics (RACH), % (*N*)	Acute assessment (ARI), % (*N*)	Emergency department (ARI), % (*N*)	Acute assessment (DGH), % (*N*)
Sex					
Male	POCT Lab	53.12% (1683) 54.64% (3559)	46.62% (5092) 47.50% (2088)	52.18% (2539) 50.09% (5522)	49.78% (227) 47.16% (341)
Female	POCT Lab	46.87% (1485) 45.36% (2955)	53.34% (5830) 52.50% (2308)	47.78% (2324) 49.91% (5503)	50.22% (229) 52.84% (382)
Age group (years)					
0–4	POCT	60.42% (1914)	0.00% (0)	0.06% (3)	0.21% (1)
	Lab	69.50% (4527)	0.00% (0)	0.00% (0)	0.00% (0)
5–18	POCT	39.46% (1250)	1.1% (120)	1.28% (62)	0.44% (2)
	Lab	30.50% (1987)	0.96% (42)	1.71% (189)	0.69% (5)
19–44	POCT	N/A	14.8% (1616)	20.33% (989)	9.43% (43)
	Lab	N/A	14.79% (650)	19.98% (2203)	7.88% (57)
45–64	POCT	N/A	21.55% (2354)	28.65% (1394)	22.37% (102)
	Lab	N/A	23.84% (1048)	22.66% (2498)	15.21% (110)
≥65	POCT	N/A	62.53% (6829)	49.66% (2416)	67.54% (308)
	Lab	N/A	60.42% (2656)	55.7% (6141)	76.21% (551)

RACH, Royal Aberdeen Children Hospital; ARI, Aberdeen Royal Infirmary; DGH, Doctor Gray's Hospital; N/A, not applicable.

Age-based analysis at PAU showed that 60.42% (*N*=1914) of total POCT was undertaken in children aged 0–4 years, and 39.46% (*N*=1250) was undertaken in children aged 5–18 years. At the same unit, 69.50% (*N*=4527) of all laboratory testing was undertaken in children aged 0–4 years and 30.50% (*N*=1987) in children aged 5–18 years (Table I).

At AMIA, both POCT (62.53%, *N*=6829) and laboratory testing (60.42%, *N*=2656) for respiratory infections were performed more frequently in patients aged ≥65 years. This was also observed at AMAU, where a greater proportion of both POCT (67.54%, *N*=308) and laboratory testing (76.21%, *N*=551) was carried out in patients aged ≥65 years.

In contrast, at ED, 49.66% (*N*=2416) of all POCT and 55.70% (*N*=6141) of all laboratory testing was undertaken in patients aged ≥65 years. Interestingly, the percentage of POCT in patients aged 45–64 years was 21.5%, 28.65% and 22.37% at AMIA, ED and AMAU, respectively. The age groups which underwent the fewest tests for respiratory viruses were 19–44 years and 5–18 years for both POCT and laboratory testing (Table I).

During the study period, respiratory samples were tested using agreed clinical pathways for selecting either POCT or laboratory testing. To understand the magnitude of testing in each pathway, the number of POCT samples was compared with the number of laboratory samples. In total, 19,409 of 42,073 (46%) respiratory samples were tested using POCT across all four units, and 22,664 of 42,073 (54%) respiratory samples were tested by the laboratory pathway (Figure 1).

**Figure 1:**
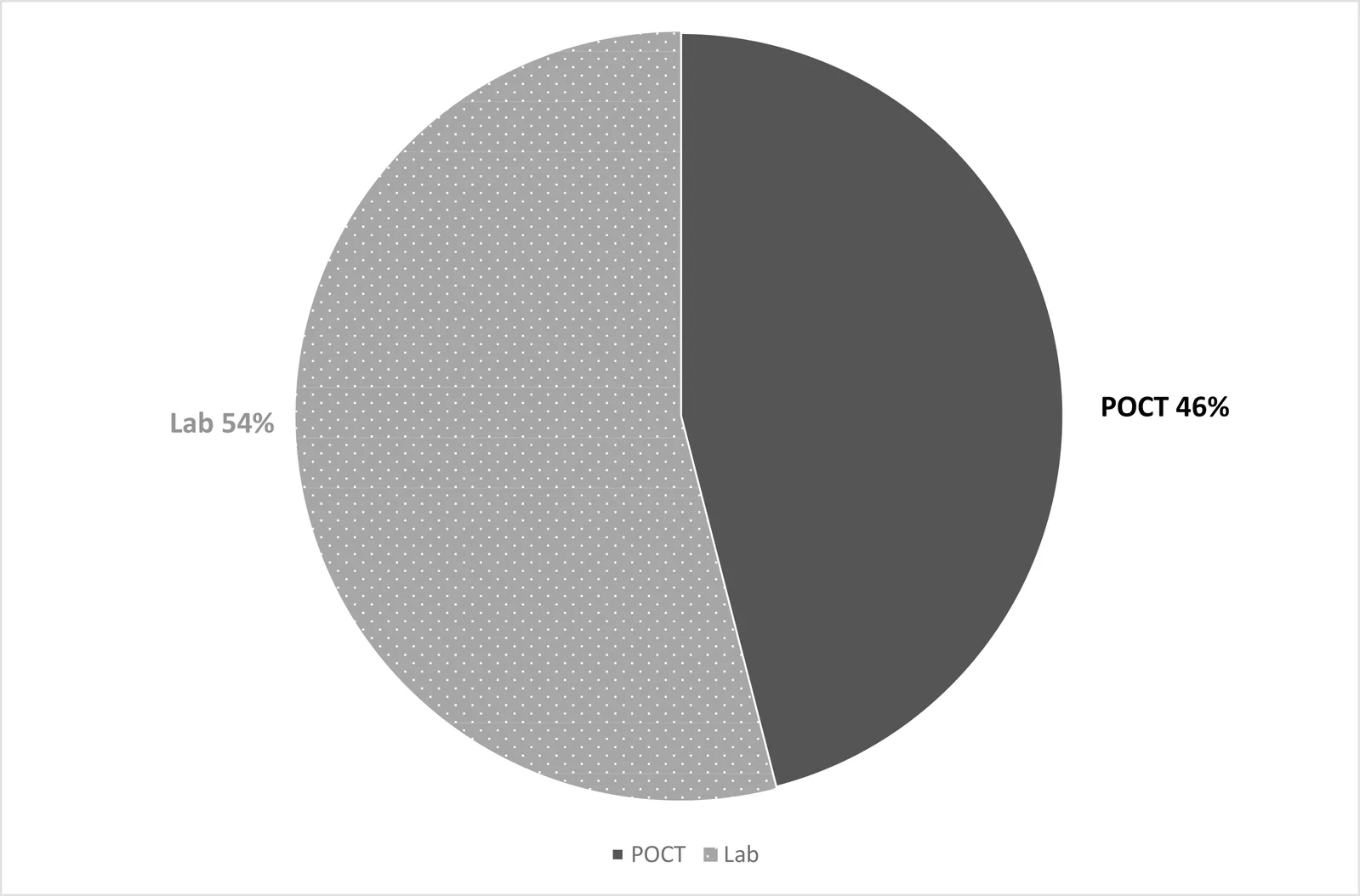
Percentage of samples tested using point-of-care testing (POCT) at the front-door settings compared with laboratory testing during the study period (January 2022–June 2024).

Location subgroup analyses showed that all four units had carried out respiratory POCT for >30% of their total samples during the study period (Table II).

**Table II:** Percentage of samples tested using point-of-care testing (POCT) for each front-door setting compared with laboratory testing during the study period (January 2022–June 2024)

Testing criteria	Paediatrics (RACH), % (*N*)	Acute assessment (ARI), % (*N*)	Emergency department (ARI), % (*N*)	Acute assessment (DGH), % (*N*)
POCT	32.72% (3168)	71.30% (10,922)	30.60% (4863)	38.68% (456)
Lab	67.28% (6514)	28.70% (4396)	69.40% (11,031)	61.32% (723)
Total	100% (9682)	100% (15318)	100% (15,894)	100% (1179)

RACH, Royal Aberdeen Children Hospital; ARI, Aberdeen Royal Infirmary; DGH, Doctor Gray's Hospital.

Respiratory testing using the POCT pathway was highest at AMIA (71.30%, *N*=10,922) and lowest at ED (30.60%, *N*=4863).

To assess training and competency in POCT use, invalid result rates were analysed as a pragmatic indicator of operational performance in routine clinical practice. In total, 95.70% (*N*=18,589) of 19,409 respiratory samples tested using the POCT pathway gave valid results, while only 4.30% (*N*=820) were invalid (Figure 2).

**Figure 2:**
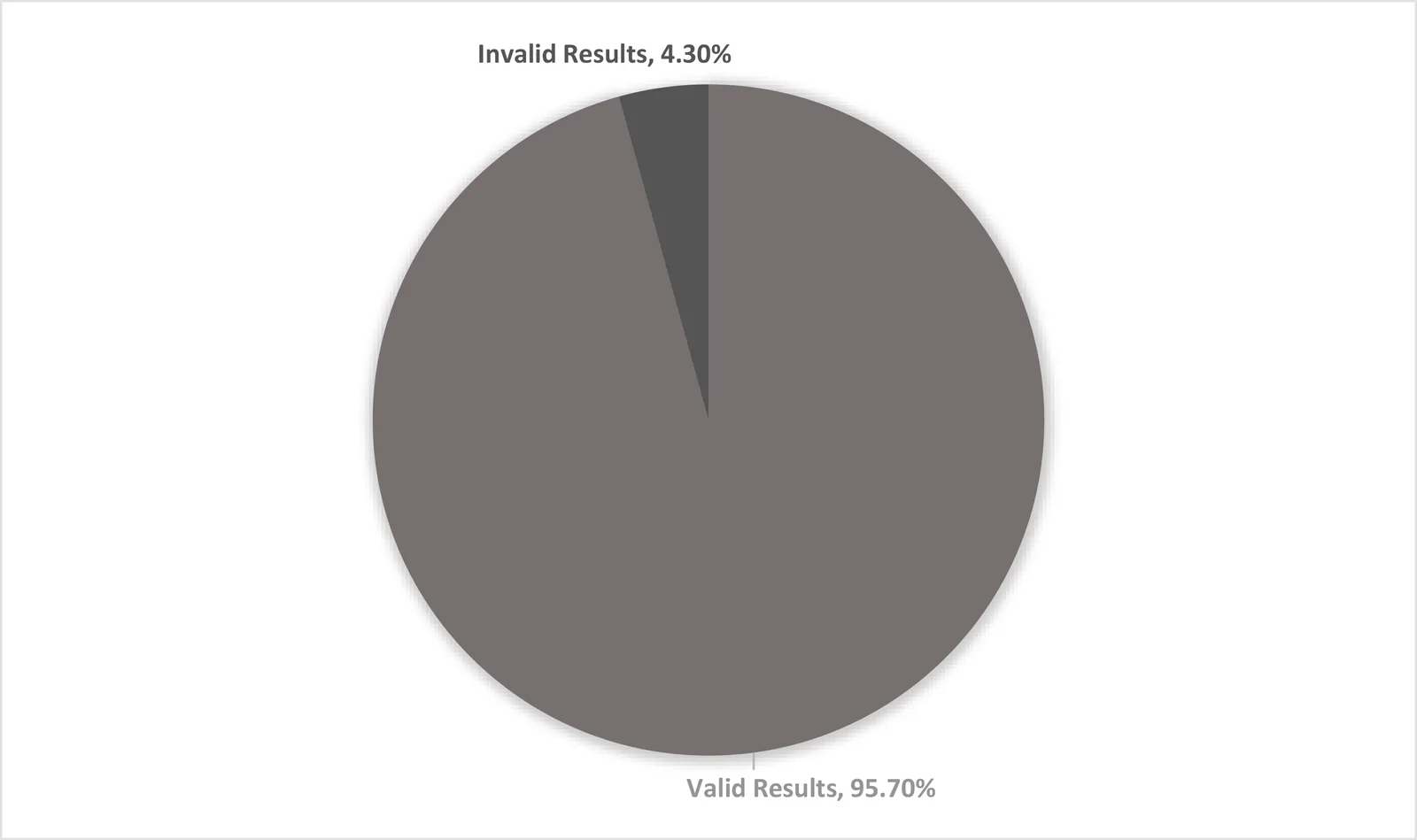
Valid and invalid results using point-of-care testing, highlighting training competency.

Additionally, subgroup analysis showed that each of the four units had >92% valid results using POCT. PAU had the lowest proportion of invalid POCT results (2.5%, *N*=79/3168), followed by AMIA (3%, *N*=333/10,922) and AMAU (4.2%, *N*=19/456). ED had the highest proportion of invalid POCT results (8%, *N*=389/4863) (Figure 3).

**Figure 3:**
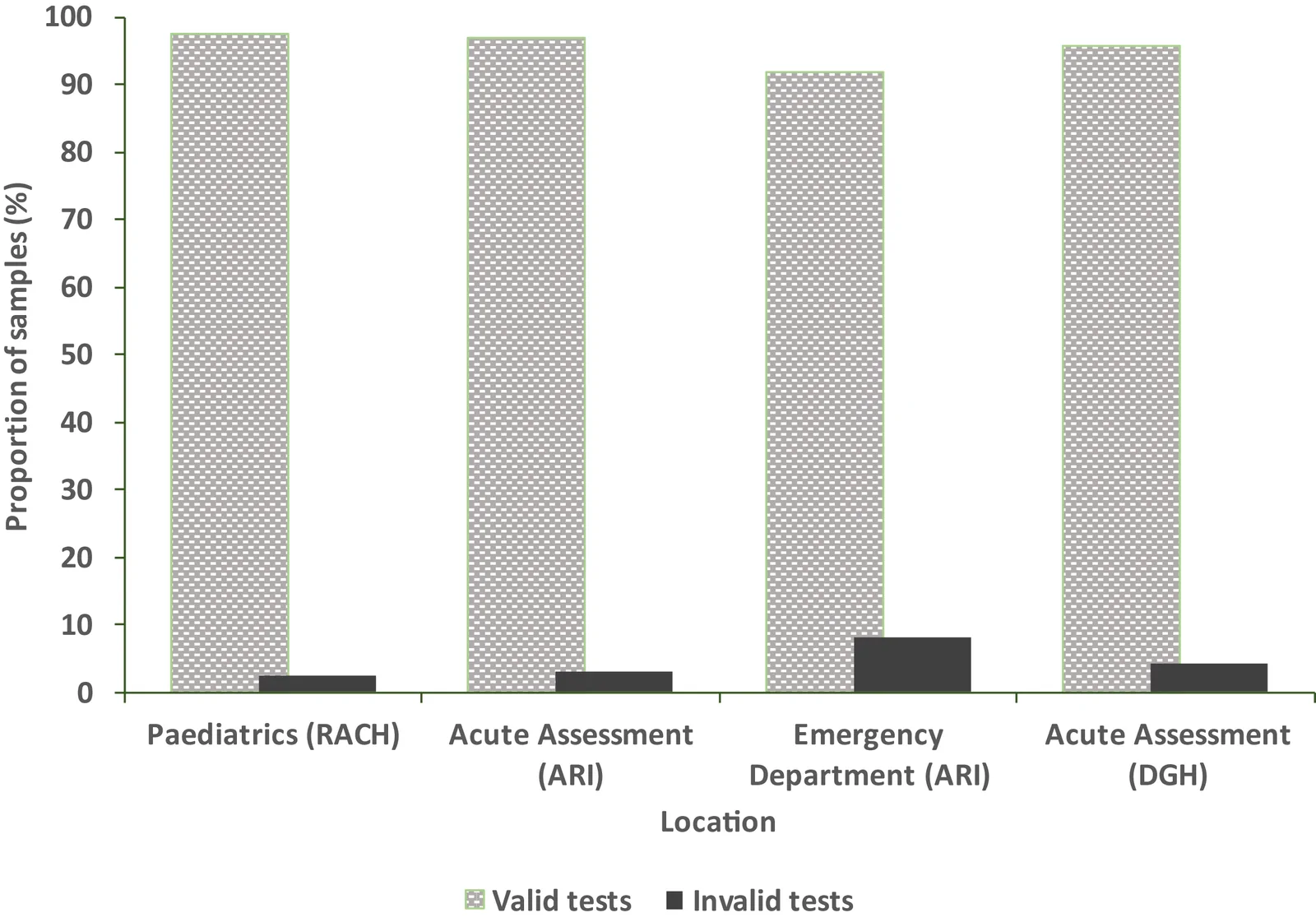
Percentage of valid and invalid results per site using point-of-care testing, highlighting its effectiveness. RACH, Royal Aberdeen Children Hospital; ARI, Aberdeen Royal Infirmary; DGH, Doctor Gray's Hospital.

Laboratory TAT was 1319 and 418 min for influenza A/B and SARS-CoV-2 testing, respectively. In contrast, for the 19,409 samples tested at the four units using POCT for influenza A/B and SARS-CoV-2, the TAT was 20 min. The difference in TAT between POCT and laboratory testing for influenza A/B and SARS-CoV-2 was significant (*P*<0.05) (Figure 4).

**Figure 4:**
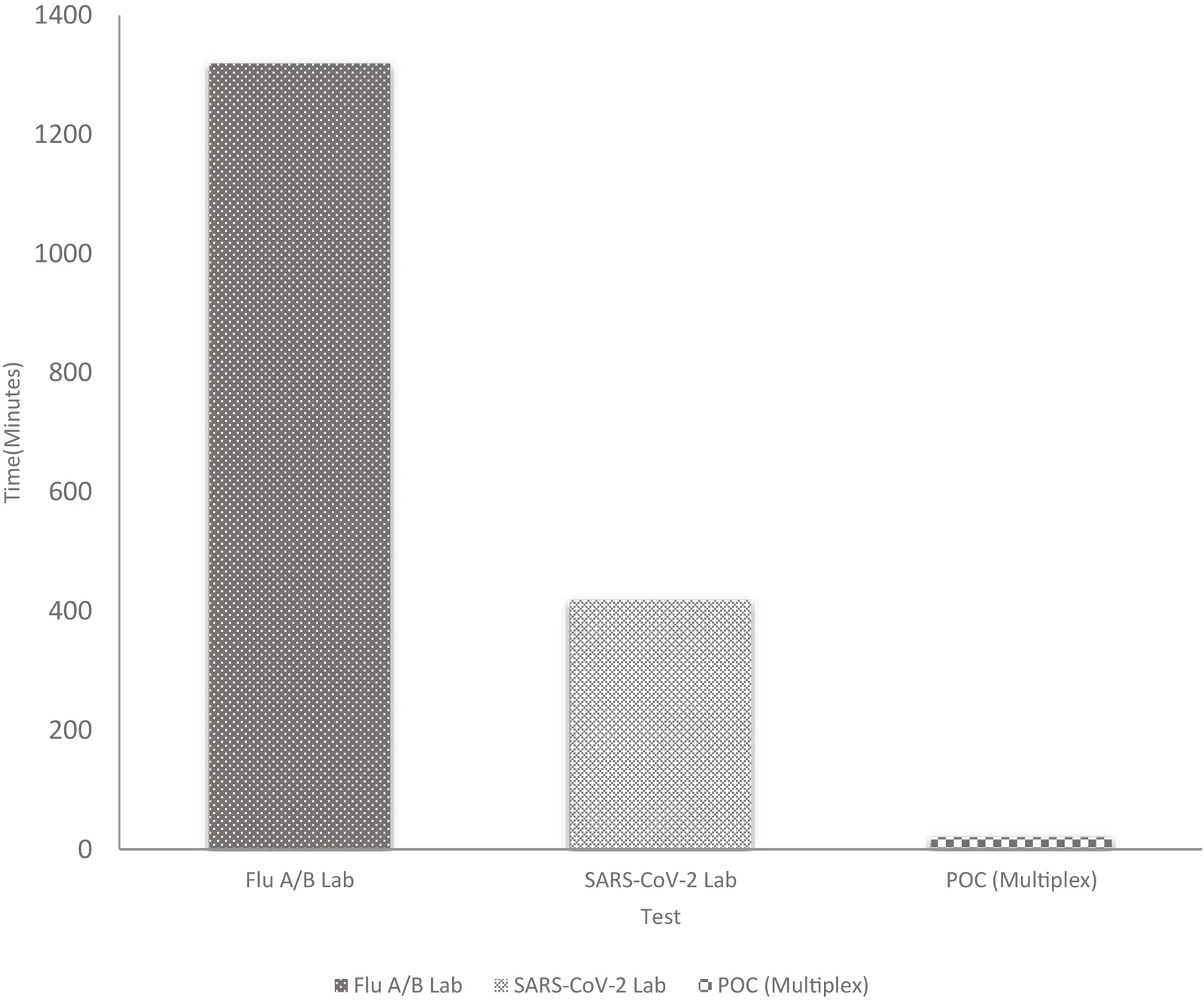
Comparative analysis of turnaround time for laboratory and point-of-care testing (POCT) testing for influenza A/B virus (Flu A/B) and severe acute respiratory syndrome coronavirus 2 (SARS-CoV-2). Laboratory and POCT turnaround times represent different workflow intervals, and therefore should not be interpreted as directly equivalent measurements.

Data were analysed to explore if there was seasonality for positive influenza A and SARS-CoV-2 results. The monthly positivity rate for influenza A showed that there were two main peaks for influenza A positivity. The peaks aligned with the winter season, with a rise in October, peaking in December/January, and a fall in March. This was observed in 2022 and 2023. There was no difference in influenza A seasonality between the four front-door assessment units, except location-specific peaks at PAU in June–August 2023 and AMAU in July–September 2023 (Figure 5).

**Figure 5:**
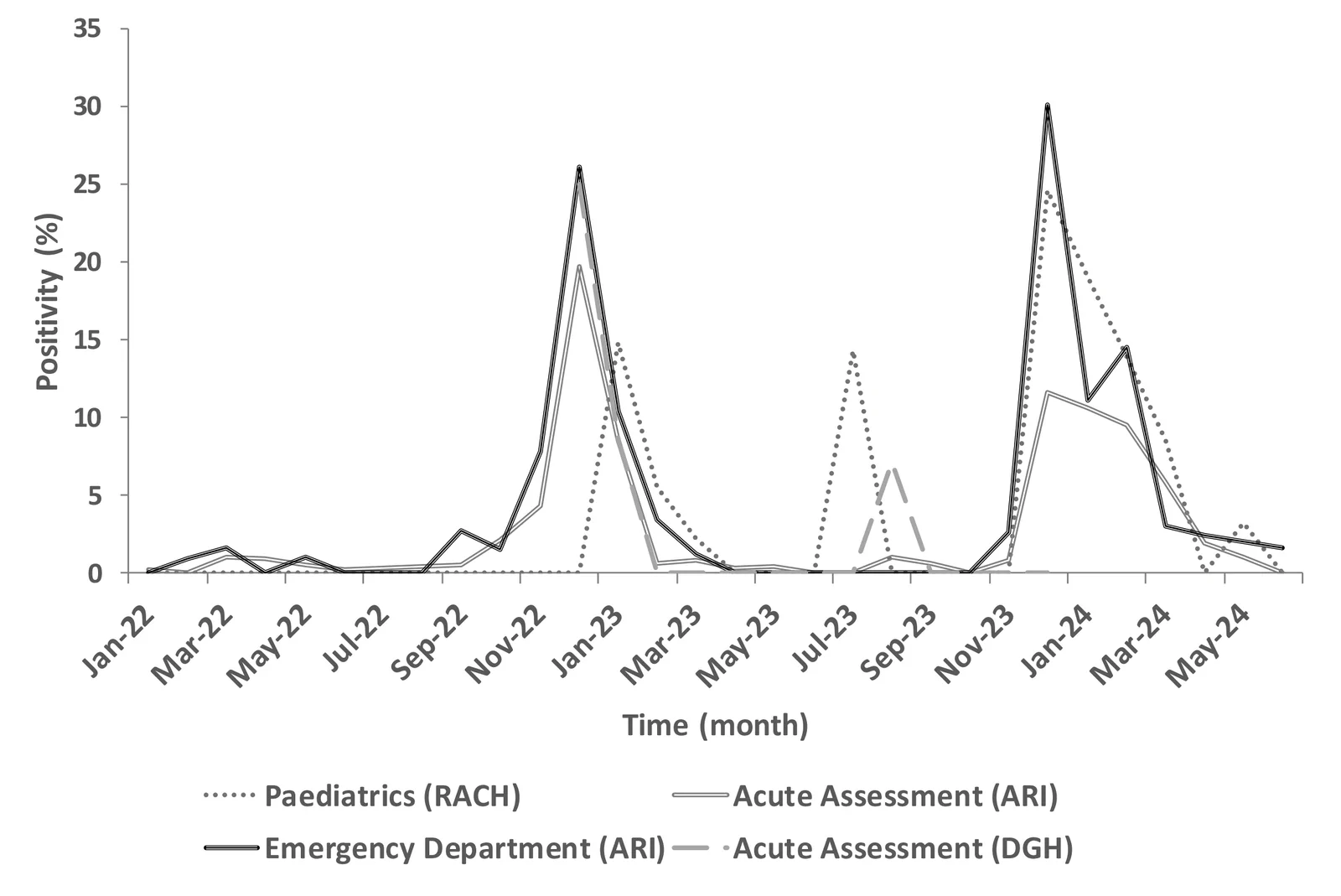
Monthly positivity rates for influenza A virus at the four front-door assessment units. RACH, Royal Aberdeen Children Hospital; ARI, Aberdeen Royal Infirmary; DGH, Doctor Gray's Hospital.

SAR-CoV-2-positive results during the study period showed no seasonal variation at any of the four units. Similarly, no location-specific positivity was observed for SARS-CoV-2 during the study period (Figure 6).

**Figure 6:**
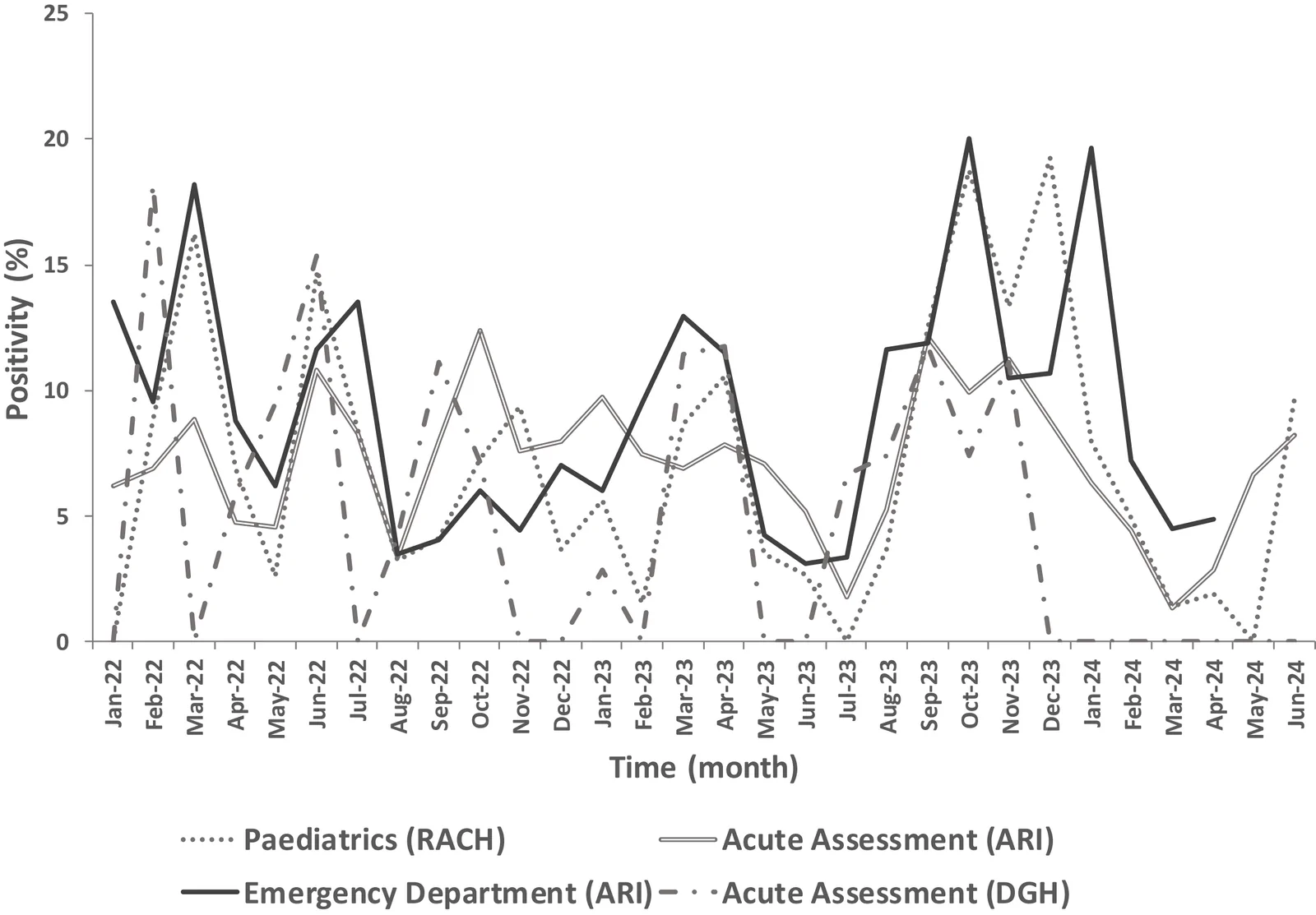
Monthly positivity rates for severe acute respiratory syndrome coronavirus 2 at the four front-door assessment units. RACH, Royal Aberdeen Children Hospital; ARI, Aberdeen Royal Infirmary; DGH, Doctor Gray's Hospital.

Age-based analysis shows that the highest rates of influenza A positivity were seen in the age groups 0–4 years and ≥65 years, with positivity rates of 69% and 43%, respectively. Similarly, the highest rates of SARS-CoV-2 positivity were observed in these age groups (72% and 63%, respectively). Patients aged 5–18 years had the lowest rates of influenza A (2%) and SARS-CoV-2 (0.6%) positivity during the study period (Figure 7).

**Figure 7:**
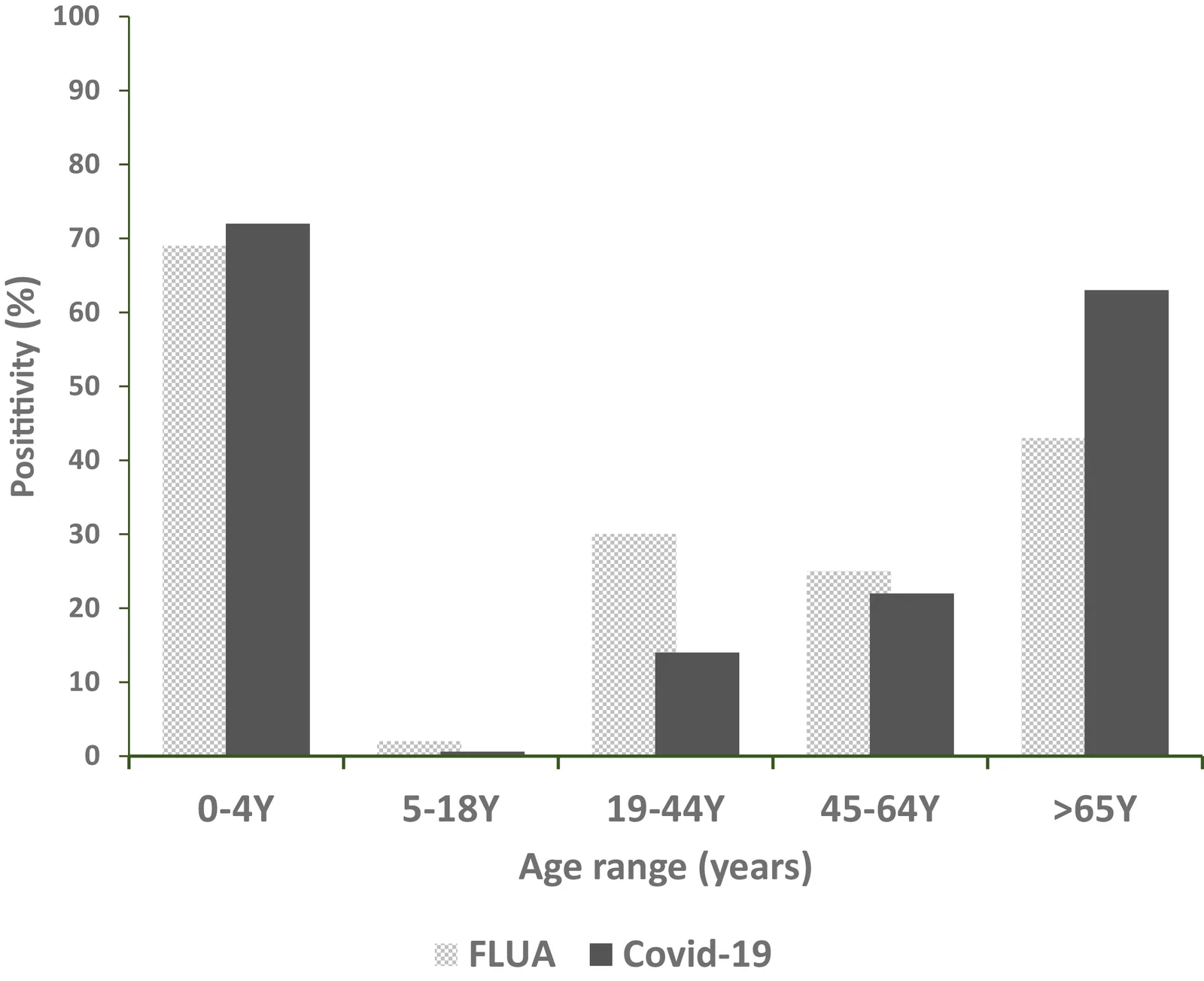
Age-based analysis of point-of-care testing positivity rates for influenza A (FLUA) and severe acute respiratory syndrome coronavirus 2 (Covid-19) at the front-door assessment units.

## Discussion

ARTIs pose a substantial global health burden, particularly during winter months, with high morbidity and mortality among vulnerable populations such as young children and the elderly. The COVID-19 pandemic exemplifies the severe impact of respiratory infectious diseases, which strain healthcare systems worldwide. A major clinical challenge lies in distinguishing between bacterial and viral infections due to overlapping symptoms [[10], [11], [12]]. In this context, POCT has emerged as a critical component in specific clinical pathways, particularly for patients presenting to hospital settings with respiratory distress. While not every patient requires POCT, its targeted implementation supports accurate and timely diagnosis, informs appropriate clinical decision-making and appropriate patient placement, and enhances patient management and public health outcomes [[12], [13], [14], [15],^21^,^22^,^26^].

Respiratory testing typically increases during the winter months due to the seasonal surge in respiratory viral infections, particularly influenza. Understanding the extent to which this seasonal demand impacts laboratory capacity is crucial. As such, one of the primary aims of this study was to explore how POCT could help alleviate the pressure on the virology laboratory during these peak periods. This analysis examined the total number of respiratory samples tested via POCT in relation to the overall number of respiratory samples received over the study period. Notably, 46% of all respiratory samples were tested using the POCT pathway across the four front-door assessment units. This substantial proportion highlights the significant role that POCT played in managing testing demand, and demonstrates how POCT contributed meaningfully to reducing the demands for laboratory testing. In addition, the high proportion of POCT made a considerable contribution to respiratory virus testing on these wards, particularly during periods of high demand such as the winter season. To the best of the authors' knowledge, this is the first study to investigate the impact of POCT for respiratory viruses on laboratory workload and testing demand. Mahieu *et al.* [33] examined the effect of POCT for blood analyses on laboratory testing and workload. Their retrospective study over a 4-year period in a neonatal intensive care unit in Antwerp, Belgium assessed the implementation of POCT blood analysers. Their findings indicated a significant reduction in the number of laboratory-processed samples (32% for bilirubin and 38% for electrolytes) following introduction of the POCT system. Given the limited research addressing the impact of respiratory POCT on laboratory workload, further investigation is needed to explore how POCT can support laboratory operations and alleviate pressure, particularly during peak periods of respiratory virus transmission, when the demand for diagnostic testing is at its highest. In addition, this shift in testing may also have allowed the laboratory to refocus resources towards more complex diagnostic assays and specialized tasks, although this was not evaluated formally in this study.

To the best of the authors' knowledge, this study is among the few to evaluate operational performance of respiratory POCT across multiple front-door clinical settings, including the proportion of valid and invalid results from influenza A/B and SARS-CoV-2 POCT, providing insight into the effectiveness of staff training and competency across four clinical sites. More than 95% of POCT results were valid, while 4.30% were invalid, demonstrating reliable implementation despite testing being performed by non-laboratory-trained clinical staff in high-throughput environments.

The structured training framework, including supervised practice, competency assessment, and ongoing governance through ward-based POCT champions and laboratory oversight, likely contributed to the high proportion of valid results observed across sites. Site-specific analysis demonstrated consistently low rates of invalid results, although a relatively higher proportion was noted for ED, potentially reflecting higher patient turnover and greater clinical complexity.

While invalid result rates provide a useful, pragmatic indicator of operational performance and user competency, it is important to recognize that such results may arise from multiple factors, including device-related issues, sample quality, or procedural variation, and should not be attributed solely to operator performance. Furthermore, temporal trends in invalid POCT rates were not formally analysed due to the relatively small number of invalid results. These findings nevertheless support the feasibility and reliability of POCT implementation in routine clinical practice when supported by appropriate training and governance structures.

It is noted that ED had the lowest utilization rate of POCT compared with laboratory testing across all sites, and also had a higher rate of invalid test results. This suggests that, although POCT remained beneficial at ED, its effectiveness in this high-pressure environment may be comparatively lower compared with other clinical areas such as AMIA and PAU. Given these insights, and particularly in settings with limited resources, these data may support prioritizing the deployment and supply of POCT to areas such as AMIA and PAU, where usage is higher, and results are more consistently valid.

This targeted approach may help to optimize resource utilization, and maximize the clinical value of POCT in front-line healthcare delivery. Moreover, these findings demonstrate that training provided to clinical staff was effective in achieving staff competency for POCT usage. To maintain high rates of valid results and reduce the incidence of invalid tests, the four sites implemented rigorous procedures including regular review of policies and protocols, and training programmes were continuously monitored and updated. Designated laboratory personnel were responsible for training clinical staff on key procedures, including sample testing, result documentation, waste disposal, and competency assessment. Only individuals who achieved competency were authorized to conduct POCT. This was explored by Yenice *et al.* [34], who highlighted the importance of structured training and competency assessment strategies in achieving optimal POCT outcomes. Most existing literature has focused on the analytical performance of POCT, including sensitivity and specificity, rather than the role of operator competency. Gibson *et al.* [18] studied the performance of the Cobas LIAT Influenza A/B and RSV assay, reporting sensitivity of 99.6%. Their analysis showed that 98.2% of patient specimens yielded valid results, with only 1.8% being invalid. Upon repeat testing of invalid samples, the proportion of valid results increased to 98.7%, further affirming the robustness and diagnostic utility of the assay. Similarly, other studies have confirmed the high sensitivity and accuracy of respiratory POCT in detecting infection [35,36]. Further research is needed to assess training effectiveness and staff competency to inform best practices in implementation of POCT in clinical settings.

This multi-site study demonstrated a significantly shorter TAT for POCT compared with laboratory-based testing for influenza A/B and SARS-CoV-2 in all four units. The shorter TAT provides an important tool that may support timely clinical decision-making, patient placement and antibiotic stewardship, and improve overall patient management. These findings align with those reported by Pedersen *et al.* [27], who evaluated implementation of the Cobas LIAT system in the ED at a tertiary care hospital. They demonstrated a reduced TAT following the introduction of respiratory virus POCT, and suggested that rapid and accurate respiratory POCT could enhance infection control, optimize antiviral and antibiotic use, and reduce hospital length of stay.

Similarly, in a multi-centre study across four EDs, including one paediatric ED in Denmark, Schneider *et al.* [29] assessed the impact of POCT for respiratory infections by comparing outcomes based on positive vs negative results. Their data showed that POCT improved antiviral treatment initiation, optimized antibiotic prescription and duration, and contributed to shorter hospital stays. However, the impact of POCT on antibiotic use and length of hospital stay is multi-factorial and remains debated. Busson *et al.* [28] evaluated the FilmArray respiratory panel in both paediatric and adult EDs in Brussels, and despite a reduced TAT, POCT did not significantly influence antibiotic prescribing practices, decisions regarding hospitalization, or the length of hospital stay.

One potential limitation of this study is that the definition of TAT differed between the laboratory tests (time from sample receipt at the laboratory to result reporting) and POCT (time to result availability following processing on the POCT device). While these definitions reflect different stages within the diagnostic pathways, both include pre-analytical and analytical components. However, the pre-analytical steps of the POCT pathway are inherently minimal given the nature of this workflow, the location of the machine next to the patient's bed, the fact that testing is performed on demand at any time point with no batch testing, and the fact that testing is performed by a single dedicated member of staff.

Factors beyond respiratory virus diagnostics, such as the results of other laboratory tests, play a role in clinical decision-making and patient flow. Although the evaluation of patient flow was beyond the scope of this study, the impact of POCT implementation on downstream clinical outcomes such as bed management, isolation room utilization, and hospital-acquired infection rates was not formally assessed. Anecdotally, implementation of POCT at the front-door assessment units may have facilitated more timely and informed clinical decisions, enabled earlier initiation of infection control measures, especially during the COVID-19 pandemic; however, these outcomes were not measured quantitatively. Additionally, the use of POCT at front-door assessment units likely supported antibiotic stewardship, allowed appropriate administration or de-escalation of antiviral/antibiotic therapy where clinically indicated.

This study offered the opportunity to explore the seasonality of influenza A and SARS-CoV-2 locally, as data collection spanned a 2-year period. The monthly positivity data for influenza A revealed one seasonal peak every year, occurring at the same time. These peaks mirrored the typical winter seasonality of influenza, with an initial rise in October, peaking in December/January, followed by a decline in March. This pattern was consistent across all front-door assessment units. PAU also exhibited a peak between June and August 2023, and a similar trend was observed at AMAU between July and September 2023. These patterns may inform anticipatory planning, including scaling of POCT capacity and laboratory support during peak respiratory virus seasons.

These findings are consistent with national surveillance data from Public Health Scotland (PHS), which demonstrated a similar trend during weeks 51 and 52 of the 2022/2023 and 2023/2024 respiratory virus seasons [37]. Factors contributing to the winter peak in respiratory viruses could include viral properties (e.g. stability and transmission mode), environmental factors (e.g. fluctuations in temperature and humidity), and host-related factors (e.g. immune response and behavioural patterns). The study findings highlight the need for robust winter planning, flexible testing, and a timely resource-allocation system capable of adapting to seasonal demand across different settings.

In contrast to influenza A, SARS-CoV-2 did not exhibit a consistent seasonal pattern in this study. Across the 2-year period, variable positivity rates and peaks were observed, with no clearly defined seasonal trend. The findings are consistent with national surveillance data from PHS, which demonstrated a similar trend for the 2022/2023 and 2023/2024 respiratory virus seasons [37]. A literature review by Townsend *et al.* [38] looked at SARS-CoV-2 transmission compared with other human coronaviruses that share similar modes of transmission, and concluded that identifying a clear seasonal trend for SARS-CoV-2 remains challenging due to multiple factors including geographic heterogeneity and the effect of differences in climate and environmental conditions.

The absence of population-level immunity during the early stages of the COVID-19 pandemic, and the emergence of new SARS-CoV-2 variants have also been proposed as other factors that allowed prolonged viral transmission [39,40]. This variability highlights the importance of maintaining a robust, responsive healthcare infrastructure capable of adapting to high demand throughout the year.

Given the current gaps in understanding the mechanisms driving the seasonality of respiratory viruses, particularly SARS-CoV-2, further research is essential to support more effective global public health planning and preparedness for future pandemics.

This study included both adult and paediatric populations, and the data revealed that the highest positivity rates for influenza A and SARS-CoV-2 were observed in two distinct age groups: children aged <5 years and adults aged ≥65 years; this finding is consistent with the Scottish national epidemiological data [41]. As POCT was applied based on clinical need rather than predefined age criteria, the age-related patterns identified likely represent real-world patient presentation and clinical decision-making in front-line admission settings, although differences in case-mix between POCT and laboratory pathways should be considered when interpreting these findings. These high-risk populations are particularly susceptible to respiratory infections, necessitating targeted healthcare resource allocation and tailored clinical management strategies. The present findings are consistent with the results of Cromer *et al.* [42], who found that children aged 6 months to 4 years exhibit the highest rate of hospitalization for influenza-related illness, while the highest case fatality rate was observed among patients aged ≥65 years [38]. These findings have important implications for service planning, including targeted deployment of POCT, prioritization of high-risk patient groups, and resource allocation during periods of increased seasonal demand.

While the cost analysis of POCT falls outside the scope of the present study, it is important to acknowledge its broader economic implications. Although POCT is generally more expensive per test compared with conventional laboratory PCR testing, it has the potential to result in significant cost savings at the system level. By enabling rapid and timely clinical decision-making, POCT can reduce hospital admission costs [20,31,32]; shorten the length of hospital stay [21,22]; decrease the need for isolation rooms [30], including staff allocation to these rooms; and reduce unnecessary antibiotic prescriptions [[24], [25], [26]]. These factors could contribute to improved efficiency and reduce overall healthcare costs.

Furthermore, the widespread use of POCT may have facilitated timely clinical decision-making, improved patient flow, enhanced infection control measures, and supported better overall patient management. Therefore, while the initial cost of POCT may be higher, the long-term clinical and economic benefits justify its integration, particularly in high-demand settings.

### Limitations and strengths

This study was conducted in Northeast Scotland, and no comparative analysis with other health boards was performed; this may limit the broader applicability of the findings. Moreover, the absence of detailed data on invalid POCT results restricted the ability to distinguish between operator-related and device-related causes. The study did not include assessment of clinical outcomes such as admission rate, respiratory complications, morbidity and mortality, particularly within the two most affected age groups: children aged <5 years and adults aged ≥65 years. In addition, the POCT multiplex panel used did not include respiratory syncytial virus testing, which may limit its clinical utility in paediatric populations.

A key strength of this study lies in its comprehensive evaluation of POCT implementation across four distinct front-door clinical settings, each serving a wide demographic spectrum. This multi-site approach offers valuable insights into the feasibility, performance and clinical integration of POCT in varied, high-throughput healthcare environments.

In conclusion, the use of POCT (46%) highlights its considerable contribution to SARS-CoV-2 and influenza A/B testing in acute and emergency admission wards, especially during periods of high demand such as the winter season.

Further studies are needed to evaluate innovative strategies and implementation models for optimal integration of POCT into healthcare systems. Such research should focus on how POCT can support laboratory services and reduce operational strain, especially during peak respiratory virus seasons when demand for diagnostic testing is at its highest.

This study demonstrates strong clinical confidence in POCT, reinforcing its diagnostic value for clinical teams, and the ease of use with high competency of staff. This can help inform targeted strategy and prioritization of resources in front-line healthcare delivery.

Implementation of POCT was associated with a significantly shorter TAT compared with laboratory-based testing. This has the potential to support timely clinical decision-making, optimize patient flow and placement, facilitate early infection control interventions, and enhance antimicrobial stewardship.

Influenza A exhibits a typical winter pattern, in contrast to SARS-CoV-2 which showed no consistent seasonal trend, highlighting the need for a robust and flexible testing infrastructure, along with adaptive resource allocation strategies to manage fluctuating diagnostic demands effectively throughout the year.

Children aged <5 years and adults aged ≥65 years represent high-risk age groups, and should be prioritized in healthcare resource planning and clinical management. The study findings underscore the importance of assessing disease severity and strategically deploying POCT to support care for these vulnerable populations.

## CRediT authorship contributions statement

Noha El Sakka: Writing – review & editing, Supervision, Methodology, Conceptualization. Alzahraa Abbas: Writing – original draft, Formal analysis, Data curation, Conceptualization. D:∖MYFILES∖ELSEVIER∖INFPIP∖00100587∖au2Ijeoma Okoliegbe: Writing – review & editing, Validation, Project administration, Methodology, Investigation

## Funding sources

None.

## Conflict of interest statement

None declared.
